# CViT Weakly Supervised Network Fusing Dual-Branch Local-Global Features for Hyperspectral Image Classification

**DOI:** 10.3390/e27080869

**Published:** 2025-08-15

**Authors:** Wentao Fu, Xiyan Sun, Xiuhua Zhang, Yuanfa Ji, Jiayuan Zhang

**Affiliations:** 1 School of Information and Communication, Guilin University of Electronic Technology, Guilin 541004, China; fuwentaoaa@163.com (W.F.); jiyuanfa@163.com (Y.J.); 2 Beidou Navigation Technology Center, Guangxi Institute of Industrial Technology for Space-Time Information, Nanning 530201, China; zjy082411@163.com

**Keywords:** deep learning, feature fusion, noise suppression

## Abstract

In hyperspectral image (HSI) classification, feature learning and label accuracy play a crucial role. In actual hyperspectral scenes, however, noisy labels are unavoidable and seriously impact the performance of methods. While deep learning has achieved remarkable results in HSI classification tasks, its noise-resistant performance usually comes at the cost of feature representation capabilities. High-dimensional and deep convolution can capture rich deep semantic features, but with high complexity and resource consumption. To deal with these problems, we propose a CViT Weakly Supervised Network (CWSN) for HSI classification. Specifically, a lightweight 1D-2D two-branch network is used for local generalization and enhancement of spatial–spectral features. Then, the fusion and characterization of local and global features are achieved through the CNN-Vision Transformer (CViT) cascade strategy. The experimental results on four benchmark HSI datasets show that CWSN has good anti-noise ability and ensures the robustness and versatility of the network facing both clean and noisy training sets. Compared to other methods, the CWSN has better classification accuracy.

## 1. Introduction

Hyperspectral image (HSI) is a three-dimensional data cube with narrow spectral bands and continuous spectral curves. It has the advantage of merging images and spectrum, resulting in superior spectral discrimination. Compared to remote sensing data such as panchromatic and multispectral images, HSI offers more significant advantages in identifying accurate subtle differences of objects, extracting complex characteristics of objects, and providing detailed spectral information of objects. HSI classification is a critical part of the application of hyperspectral remote sensing technology, and its classification results have important application value in humanities and natural domains, such as fine agriculture [1,2], resource exploration [3,4], and ecological monitoring [5,6].

With the continuous update of hyperspectral sensors, the corresponding image processing and information classification and extraction methods have also advanced significantly [7,8,9,10]. HSI interpretation in the supervised paradigm is divided into two phases: feature extraction [11,12] and object classification. Among them, the feature extraction stage progresses from using spatial or spectral characteristics alone to combining spatial and spectral features [13,14,15,16,17,18,19]. In terms of classification algorithms, the most representative traditional machine learning methods include decision tree classification [20,21], support vector machines [22,23], sparse representation [24,25], and multiple classifier systems [26,27]. However, they mainly rely on manually designed shallow features, and their performance is limited for data with complicated nonlinear relationships and rich feature information. Deep learning methods, with their robust hierarchical feature representation capabilities and autonomous learning qualities, broaden the application scope in remote sensing. CNNs, for example, offer a wide range of effective solutions for HSI classification. Three-dimensional CNNs can perform joint spatial–spectral feature extraction, and with the increase in the number of layers and the number of convolutional kernels, they can capture rich deep semantic features, which is correspondingly accompanied by higher computational resources, time costs, and training sample requirements. Convolutional neural networks, such as group convolution and depthwise separable convolution, can retain their performance while reducing the number of parameters. Due to their limited receptive field, traditional CNNs are unable to learn the global contextual information of the HSI cube. To address the issue of a limited receptive field, researchers have proposed several methods to improve the understanding of global information by CNNs. One method is to increase the number of network layers, which can naturally expand the receptive field. However, the inherent problem of vanishing or exploding gradients was not overcome until the emergence of residual networks (ResNets). The residual connection not only makes the construction of deep networks possible, but also provides a better training environment for the network, ensures the effective transfer of information and the stability of the training process, and improves the expressiveness and overall robustness of the model. Second, the size and structure of the convolution kernel are designed for feature fusion at different scales, as seen with the introduction of inflated convolution and the inception module. Third, long-range information transfer and global dependence modelling are achieved by calculating the interactions between any two positions in the input feature map, such as the introduction of non-local blocks, spatial–spectral dual-attention strategy, and self-attention mechanism. In particular, the Transformer [28,29,30], a representative model of the self-attention mechanism, has been gradually extended and derived into the field of computer vision following its achievement in Natural Language Processing (NLP) due to its powerful modelling capability. For example, Vision Transformer (ViT), CNN-Transformer, inherits the core components of Transformer on top of CNNs and uses the multi-head self-attention (MHSA) mechanism to allow each position to interact with all the other positions, helping the model to better understand the local details and global structure of the HSI cube.

These classification methods based on supervised paradigms need to be based on high-confidence training samples [31,32,33,34]. However, due to the influence of environmental interference and manual labeling errors, noise samples are unavoidable in the training set, which seriously affects the performance of the classification method. Therefore, related research has been carried out, for example, designing deep learning network architectures to improve robustness, but deep or high-dimensional convolution suffers from high resource consumption and optimization difficulties. The noise label removal strategy tends to over-clean the dataset, greatly reduces the number of training samples, and also results in the loss of real samples and feature information.

To improve the information suppression ability of semantic feature extraction and noisy training samples for deep learning in HSI classification, this paper proposes a CViT Weakly Supervised Network (CWSN). First, the model creates a spectral and spatial information deep extraction network using one-dimensional and two-dimensional convolutional functions, and then efficiently fuses the shallow features with the high-level features by residual connections to alleviate the gradient vanishing problem, and further enhance the expression of discriminative spatial spectral features. The spatial–spectral features are then processed using CNN-Vision Transformer (CViT) multi-head self-attention to capture the long-range dependence between image elements. In addition, a noise suppression loss function is created using the cross-entropy loss function theory to effectively guide the network in learning and utilizing the prior knowledge. The main contributions of this work are listed below.

(1) In this paper, we propose a CWSN that integrates a lightweight dual-branch feature enhancement module and a CNN-Vision Transformer, while organically integrating deep semantic feature extraction and noisy sample processing into a deep learning framework.
(2) A Dual-Branch Local Induction Module (DBLIM) is designed, which has a simple architecture, a small number of parameters, and a high generalization capacity. This module can enhance the discriminative and divisible nature of different classes of feature information, and mitigate the gradient vanishing of the depth model.
(3) Local and global deep semantic features are generalized and characterized using CViT, together with Noise Suppression Loss (NSL), which enhances the robustness of the model and makes it stable in the face of both clean and noisy training sets.

The rest of the paper is organized as follows. In Section 2, some related work is presented. Section 3 describes the proposed algorithm in detail. Section 4 includes the design of the model components, a comparative evaluation of the classification performance, and an application analysis. Finally, conclusions are drawn in Section 5.

## 2. Related Work

### 2.1. HSI Supervised Classification

Over the last two decades, there have been numerous classification methods used in remote sensing image classification research, ranging from manual visual interpretation to manual repair based on automatic computer classification to automatic computer classification using supervised and unsupervised algorithms. In particular, new machine learning and deep learning methods have evolved in recent years, allowing for significant advances in classification accuracy and efficiency, with HSI classification mixed with artificial intelligence algorithms serving as the current research frontier. Trias-Sanz et al. [35] summarized and investigated the combination of color and texture features using quantitative evaluation of hierarchical segmentation algorithms to achieve the best results for the task of segmentation of high-resolution multispectral aerial images in rural areas. However, the use of spatial features alone does not apply to lower spatial resolution remote sensing image classification. Xia et al. [36] proposed an integrated method, Rotation Random Forest via KPCA (RoRF-KPCA), in which KPCA extracts information from each subset of the original feature space, and the resulting feature sets are merged and fed into the RF classifier. However, traditional spectral classifiers have low classification accuracy values for feature classes with similar spectral responses. Therefore, many researchers began to develop extraction and optimization techniques using spatial–spectral features, with deep learning being the most representative. Sun et al. [37] proposed Spatial–Spatial Feature Tokenization Transformer (SSFTT) to extract shallow spectral and spatial features through a designed convolutional module and transformed them using Gaussian-weighted feature tokenization. He et al. [38] proposed the Spatial–Spectral Transformer (SST) to extract spatial and spectral features, with a multilayer perceptron performing the final classification task. HSI classification methods using spatial–spectral features have achieved considerable results on reliable training datasets; however, these methods lack robustness when dealing with noisy labels.

### 2.2. HSI Weakly Supervised Classification

In order to mitigate the impact of noisy labels on HSI classification, researchers have successively developed solutions from different perspectives and proposed a noisy label learning strategy.

For the correction of noisy labels, Kang et al. [39] developed a mislabeled sample detection and correction method based on edge preserving filtering and spectral constraints, which utilizes spectral constraints to identify and correct mislabeled samples in training samples while preserving the edge features of the image. Li et al. [40] proposed the Adaptive Selective Loss Propagation (ASLP), which first identifies an adaptive number of samples with lower loss values from the untrusted set to supplement the trusted set, and then performs label propagation based on the enlarged trusted set to achieve label correction. Leng et al. [41] designed the Sparse Graph-Based Adaptive Label Propagation (SALP) algorithm, which relies on the assumption that the noise has a Gaussian distribution and provides an effective mechanism for detecting noisy labels in the training set. The above label correction methods perform better in simple noise environments (with random noisy labels), and all of them are combined with machine learning methods to complete the classification. It remains to be investigated if they are applied to complex noise environments or deep learning.

For noisy label removal, Tu et al. [42] first developed a Density Peak-based Noisy Label Detection (DPNLD) method that detects and removes noisy labels based on distance and local density. Later, a noisy label detection framework [43] was proposed, which combines Superpixel-to-Pixel Weighting Distance (SPWD) and density peak clustering to accurately detect and remove noisy labels from the training set before HSI classification. Fang C et al. [8] proposed a deep reinforcement learning framework method for learning each label’s correctness and removing different types of label noise. Class methods for noisy label removal tend to over-clean the dataset, resulting in the loss of real samples and feature information.

In terms of noisy label suppression, the robustness of the classifier is mainly improved by improving the structure of the deep learning network, creating the anti-noise loss function, and evaluating the label confidence, etc. To extract the spectral spatial features, Ghaderizadeh et al. [44] combined a 3D fast-learning block and a 2D convolutional neural network. This 3D hybrid convolutional neural network (3DCNN) reduces the model complexity while also being effective against noise and a limited number of training samples. Wang et al. [45] proposed a Dual Spectral–Spatial Residual Adaptive (DSSRA) network with an Adaptive Noise-Robust Loss (ANRL) function in the network to mitigate the effects of learning with noisy labels. Zhang et al. [46] proposed a Triple Contrastive Representation Learning (TCRL) framework from a deep clustering perspective to learn feature information while reducing classifier overfitting to noisy labels. Yue et al. [47] developed a novel spectral-feature-based HSI multi-source label noise simulation method that evaluates the overall credibility of different label sources and the credibility of specific categories, aiming to mitigate the impact of multi-source noisy labels. To improve robustness, noise suppression methods reduce the representational capacity of its high-level semantic features, which degrades the classification performance when dealing with credible datasets.

Compared to traditional shallow machine learning methods, deep learning training requires a larger number of high-confidence training samples, making sample mislabeling a more serious issue. Therefore, while improving the ability of the deep learning model to cope with noise, it is important to achieve the full extraction and utilization of spatial–spectral information, as well as to ensure high interpretation accuracy even on small and reliable sample sets.

## 3. Proposed Model

**Figure 1:**
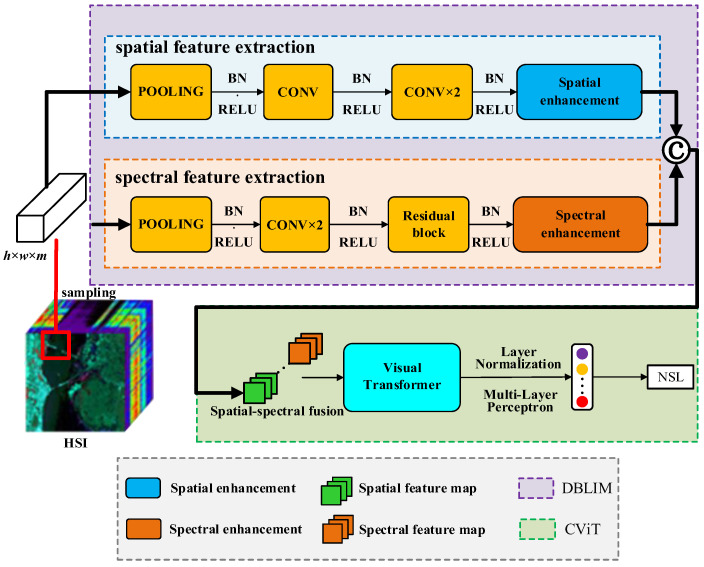
Framework of the CWSN model.

We created CWSN, a novel noise suppression HSI classification model, which aims to achieve deep extraction and characterization of spatial spectral features locally and globally, as well as mitigating the effect of noisy training sets on HSI classification. Figure 1 shows the model framework, which is made up of two modules, DBLIM and CViT, and incorporates the NSL. Since the training samples for HSI classification are usually cubic in format, several 3D cubes are randomly selected from the HSI as inputs to the CWSN, and the cubes are used as an example to illustrate the proposed network structure.

### 3.1. Dual-Branch Local Induction Module

DBLIM is used to extract deep spatial-spectral features, which consist of a 1DCNN-based spectral feature extraction channel and a 2DCNN-based spatial feature extraction channel. Unlike the standard convolution, DBLIM employs a dual-branch structure for local induction and enhancement of spatial and spectral features. In addition, residual connections are added to the spectral branches. This is because the spatial branch has a lower residual dependence due to its localization. Spectral branching has a high information dimension and strong correlations between bands; therefore, deep networks are needed to model the complex relationships and global dependencies among bands. Residual connection aids deep networks in stabilizing training, adequately extracting nonlinear features, mitigating gradient vanishing, and improving classification accuracy. Additionally, since 1D convolution has fewer parameters, residual blocks do not considerably increase the computational amount. The combination of multilayer 1D convolution and residual blocks enhances feature extraction capability and stability, and the input image block achieves a robust spectral representation while preserving the original spatial information.

#### 3.1.1. Spectral Feature Extraction Channel

**Figure 2:**
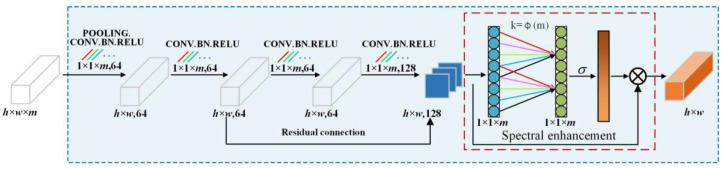
Spectral feature extraction.

The structure of the spectral feature extraction channel is shown in Figure 2, which is based on 1DCNN to achieve a lightweight design and reduce the number of parameters. The input feature $F_{in}\in R^{w\times h\times m}$ is first pooled globally on average in order to obtain a one-dimensional vector feature $F_{m}$ of size 1 × 1 × *m*. The expression of $F_{m}$ is as follow:

(1) $$F_{m}=GAP(F_{in}),F_{m}\in R^{1\times 1\times m}$$

The 1D convolution kernel is set to 1 × 1 × *m* to efficiently extract spectral features without affecting the original spatial features. This size setting ensures that features are not aggregated spatially, but cover all bands in the spectral dimension. The advantages are as follows: avoiding interference from spatial noise, such as mixed image elements, while simultaneously highlighting the spectral characteristics; and capturing global features of the spectral curve and avoiding loss of spectral continuity in segmental processing. In the first convolution layer, 64 convolution kernels of 1 × 1 × *m* size and a sampling step of (1, 1, *m*) size are used to perform a convolution operation on the input *w* × *h* × *m* 3D cube to obtain the *w* × *h* × *m* 3D cube features, eliminate redundant spectral features, and better focus on the features that are crucial in classification. Then, a residual block consisting of two consecutive convolutional layers is used for deep feature extraction and enhancement of the first layer of convolutional features, emphasizing the important regions of the image and improving the spectral robustness when dealing with the noisy labels. A batch normalization layer and an activation function are applied after each convolutional operation in order to obtain the spectral dimensional feature weighting information $\omega_{spe}$.

(2) $$\omega_{spe}=\sigma (conv1D_{k}(F_{m}))$$

where σ(⋅) and $conv1D_{k}(\cdot )$ denote the ReLU activation function and one-dimensional convolution function.

Following the residual block, the input feature is dot-multiplied with the spectral weight information to obtain the output feature $F_{out}\in R^{w\times h\times 1}$ enhanced by spectral features, which can discriminatively extract the spectral features while maintaining the separability between spectral information classes. The expression is as follows.

(3) $$F_{out}=\omega_{spe}*F_{m},F_{out}\in R^{w\times h\times 1}$$

#### 3.1.2. Spatial Feature Extraction Channel

**Figure 3:**
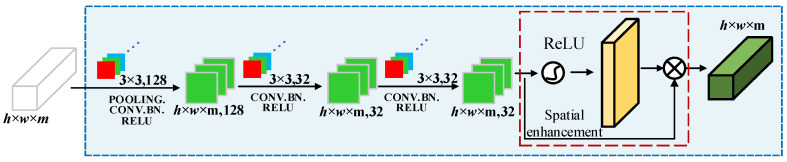
Spatial feature extraction.

For spatial feature extraction, a 2D convolution module is used to extract the spatial correlation features of the center pixel and its surrounding 8-neighborhood, as shown in Figure 3. Similarly, assuming that the input feature is $F_{in}\in R^{w\times h\times m}$, in this paper, we first performed mean pooling and maximum pooling along the spectral dimension, and performed feature stacking with the following expression:

(4) $$F_{m1}=MEANP(F_{in}),F_{m1}\in R^{w\times h\times 1}$$

(5) $$F_{m2}=MAXP(F_{in}),F_{m2}\in R^{w\times h\times 1}$$

(6) $$F_{m3}=Cat(3,\left[F_{m1},F_{m2}\right]\;),F_{m3}\in R^{w\times h\times 3}$$

where MEANP(⋅), MAXP(⋅), and Cat(⋅) denote the mean pooling operation, maximum pooling operation, and spectral dimension feature stacking operation, respectively.

The 1 × 1 convolution cannot capture spatial context information, while a larger kernel (e.g., 5 × 5) will increase the computation and lead to over-smoothing of features. Therefore, the 2D convolution kernel size is set to 3 × 3. This setting balances the receptive field and computational efficiency, and can effectively capture local spatial features (e.g., edges, points). Next, the first layer utilizes 128 convolution kernels of size 3 × 3 to perform a convolution operation on $F_{m3}$ Then, the second layer uses two convolution kernels of the same size to perform successive convolutions, and the strides are (1, 1). At the same time, the ReLU activation function is utilized to obtain the information about the spatial weights $\omega_{spa}$ of the image with the following expression:

(7) $$\omega_{spa}=\sigma (conv2D_{7\times 7}(F_{m3}))$$

Finally, the input feature is dot-multiplied with the spatial weight information to obtain the spatial feature-enhanced output feature $F_{out3}\in R^{w\times h\times m}$, so as to retain and emphasize important spatial information. The expression is as follows:

(8) $$F_{out3}=\omega_{spa}*F_{m3},F_{out3}\in R^{w\times h\times m}$$

### 3.2. CViT Hybrid Structure

#### 3.2.1. CViT

CNNs have the advantage of local feature induction, but are unable to characterize the long-range dependence, while ViT [48] has an excellent performance in long-range correlation characterization. Thus, the CNN and Transformer fusion structure can effectively capture the spatial and spectral features of HSI. Therefore, in this paper, a CViT hybrid structure was used for deep splicing and characterization of spectral and spatial features.

Spectral and spatial features are first spliced and stacked by spectral dimension. The stacked features are then projected nonlinearly using a feature mapping layer built with one-dimensional convolution, the ReLU activation function, and layer normalization. Specifically, assuming that $F_{in}\in R^{w\times h\times m}$ is the input feature of the CViT module, thirty-two convolutional kernels of size 1 × 1 are first used for the feature mapping of the input features to obtain the coded features $Y_{in}$, and its expression is shown below:

(9) $$Y_{in}=Conv1D(F_{in})$$

In order to capture the spatial long-range feature dependencies in an image block, position information $I_{pos}$ is added to the coded features $Y_{in}$ with the following expression:

(10) $${\hat{Y}}_{in}=Y_{in}+I_{pos}$$

After convolutional coding, spectral and spatial features are fused to achieve local and global deep features. A multi-head self-attention (MHSA) block, a multi-layer perceptron (MLP), and two layer normalization (LN) processes are used to model the depth relationship of the semantically tagged feature ${\hat{Y}}_{in}$. Notably, two residual connections are utilized before the MHSA block and the MLP layer to prevent information loss. In order to learn the deep semantic features, three learnable weight matrices $W_{Q}$, $W_{K}$, and $W_{V}$ are defined, and ${\hat{Y}}_{in}$ is linearly mapped to query information *Q*, key information *K*, and value information *V*, respectively, based on the above three learnable weight matrices. Then, the attention score is obtained based on the product of *Q* and *K*, and the weight of the score is obtained by the SoftMax function. The expression of the self-attention mechanism *SA* is defined as follow:

(11) $$SA=Attention(Q,K,V)=softmax(\frac{QK^{T}}{\sqrt{d}})V$$

where *d* is the dimension of *K*.

MHSA uses multiple sets of weight matrices to map *Q*, *K*, and *V* using the same process. Output projections of features are performed using modules with the same structure as the mapping layer. Thus, the final self-attention results are stacked together for output, utilizing the expression defined below:

(12) $$MHSA(Q,K,V)=Cat(SA_{1},SA_{2},\ldots ,SA_{h})W$$

where *h* is the number of heads, and *W* is a linear transformation matrix that ensures that the output feature channels are consistent.

Finally, the globally characterized features ${\hat{Y}}_{in}$ are sequentially input to the MLP layer and LN. It is worth mentioning that the MLP layer consists of two fully connected layers with an activation function in the middle. The fully connected layers are used to obtain the final class information. Furthermore, layer normalization can prevent gradient explosion or vanishing while also accelerating network convergence.

#### 3.2.2. NSL Function

This paper focuses on HSI weakly supervised classification; hence, the mechanism and definition of noisy label generation in the experiments are presented initially. For each class, *n* samples with true labels are randomly selected to form the initial training set. Then, uniformly select *m* samples from other classes and label them as the current class. After that, all the samples in each class are mixed to ensure that the noisy samples are fully incorporated into the initial training set. Then, a class C classification training set with noisy labels can be defined as $S^{c}=\left\{\left(r_{1},l_{1}^{T}),\ldots ,(r_{n},l_{n}^{T}),((r_{n+1},l_{n+1}^{T}),\ldots ,(r_{n+m},l_{n+m}^{T}\right)\right\}$, where *r* denotes the training samples and $l\in \left\{1,2,\ldots ,C\right\}$ denotes the labels of the training samples (containing noisy labels). In addition, $l^{*}$ is introduced to denote the possible correct class labels. Based on the independent homogeneous distribution assumption, there are:

(13) $$P(l=c\left|l^{*}=j,r\right.)=P(l=c\left|l^{*}=j)\right.$$

Because all samples are randomly disrupted, each label has the probability θ=m/(n+m) of being mislabeled. Therefore, the probability that sample *j* from class *i* has a potentially clean label can be expressed as:

(14) $$\left\{\begin{array}{l} \theta_{ij}=\frac{\theta }{C-1},i\neq j\\ \theta_{ij}=1-\theta ,\;\;\;\;i=j \end{array}\right.$$

where $\theta_{ij}=P(l=i\left|l^{*}=j)\right.$.

The anti-noise loss function’s central notion is to ensure that, when training on noisy samples, the optimal model parameters are also the optimal parameters when all samples are labeled correctly. To better demonstrate how the anti-noise loss function works, for the noisy training set described above, the following equation is used to describe the process of noisy label suppression:

(15) $$E_{r,l}L(f^{*}(r),l)\leq E_{r,l}L(f(r),l),\forall f\in F$$

where *F* is the possible parameter space of the model, *f* is the possible solution, and $f^{*}$ is the global minimum of the empirical risk when training with potentially clean labels $l^{*}$.

We united the normalization cross-entropy (*NCE*) loss and reverse cross-entropy (*RCE*) loss to form a noise loss function *NSL* [45]. The *NSL* is used after the MLP layer in order to effectively suppress the noisy samples in the training set and obtain the optimal weight parameters of the network model. It is worth mentioning that the *RCE* loss exchanges the positions of the predicted value $p(l\left|r)\right.$ and the true value $q(l\left|r)\right.$ in the ordinary cross-entropy (CE) loss. The expressions of *NCE*, *RCE*, and *NSL* are shown below:

(16) $$NCE(p,q)=\frac{-\sum_{c=1}^{C}q(l\left|r)\log p(l\left|r)\right.\right.}{-\sum_{j=1}^{C}\sum_{c=1}^{C}q(l=j\left|r)\log p(l=j\left|r)\right.\right.}$$

(17) $$RCE(p,q)=-\sum_{c=1}^{C}q(c|r)\log p(c|r)$$

(18) NSL(p,q)=(NCE(p,q)+RCE(p,q))/2

## 4. Experiments and Analysis of Results

### 4.1. Datasets and Experimental Setup

#### 4.1.1. Experimental Datasets

The component design, classification performance evaluation, and application analysis of the model were performed on four hyperspectral datasets using three evaluation metrics, Overall Accuracy (OA), Average Accuracy (AA), and Kappa coefficient. In the experiment, two noise label levels were set up to test the stability of the model under different noise intensities. Specifically, the low noise rate was set at about 20% for modeling light labeling noise, and the medium noise rate was set around 30% for modeling errors such as mixed pixels. The noise settings for each dataset are shown in Table 1. In addition, the experiment was repeated 10 times in each experimental environment, and the average value was used as the evaluation result.

(1) University of Pavia (UP): The ROSIS 03 sensor collected data from the University of Pavia campus in Italy. The spatial resolution is 1.3 m, with a spectral coverage of 0.43–0.86 μm. Nine classes of ground cover are included in the datasets.
(2) Washington DC (WDC): The Washington DC dataset was collected using the Hyperspectral Digital Imaging Experiment sensor over Washington DC. Seventy-eight spectral bands were selected from the 400–1000 nm spectral range to form the HSI, and the corresponding RGB images were acquired using a Sentinel-2 SRF. The dataset contains 480 scan lines with 307 pixels on each scan line.
(3) Salinas Valley (SV): The Salinas Valley dataset was collected by the Airborne Visible Infrared Imaging Spectrometer (AVIRIS), a sensor imaging the Salinas Valley region of California, USA. The spatial resolution is 3.7 m, and the number of bands is 224, covering an area with an image size of 512 rows and 217 columns, containing 16 object classes. Twenty water vapor absorption and noise bands (numbers 108–112, 145–167, and 224) have been removed from the data, leaving 204 active bands.
(4) Kennedy Space Centre (KSC): The Kennedy Space Centre dataset was collected by the AVIRIS instrument during an overflight of the Kennedy Space Centre in the USA, although with a low spatial resolution of 18 m. These data include raw spatial dimensions of 512 × 614 pixels, with 48 bands removed due to absorption and low signal-to-noise ratios, and 176 spectral bands used for analysis. It contains 13 classes representing different object classes.

#### 4.1.2. Experimental Settings

In the spatial feature extraction stage, a two-dimensional convolution function with a convolution kernel size of 3 was used to execute three consecutive spatial feature extractions, with 128, 32, and 32 convolution kernels each. The spatial attention mechanism used a convolution kernel with a size of 7. In the spectral feature extraction stage, a one-dimensional convolution function with a convolution kernel size of 1 was used to perform four consecutive spectral feature extractions, in which the numbers of convolution kernels each time were 64, 64, 64, and 128, respectively. The spectral attention mechanism’s convolution kernel had a size of 3. In addition, the number of feature dimensions, the number of multi-heads, and the feature learning depth in the ViT module were set to 32, 1, and 1, respectively.

In the training stage, this paper used the image block and its class label corresponding to the center pixel with a window size of 9 × 9 as samples to construct the training sample set. Each batch contained 16 training samples, with the learning rate set to 0.0001. In addition, this paper adopted the gradient optimization algorithm based on the Adaptive Moment Estimation (Adam) to train the CWSN. It is worth mentioning that the network hyperparameter settings in the inference phase are the same as in the training phase. The network model was run by the Pytorch framework (https://pytorch.org/) in the Windows 11 environment with 16 GB RAM and an Nvidia RTX 2080 GPU (ASUS, Shenzhen, China).

In the simulation stage, the experiment was performed 10 times in each set of experimental conditions, and finally, the average value was used as the evaluation result.

### 4.2. Parameter Sensitivity Analysis

To explore the effect of parameter variations on the performance of the CWSN model, this section conducts a sensitivity analysis of two hyperparameters in the CViT structure: feature dimension *K* and number of attention heads *h*. The parameters *K* and *h* have values in the ranges of [16, 32, 64, 128, 256] and [1, 2, 3, 4, 5]. The parameter *K* determines the dimensionality of each layer’s feature vectors inside the model, with higher feature dimensions providing richer feature representations and helping the model in capturing more information, details, and feature relationships. The parameter *h* determines the number of attention heads that are processed in parallel in the multi-head attention mechanism; different attention heads can capture different types of dependencies, such as local or global dependencies. This diversity and complementarity allow the model to gain a more complete understanding of the data. In particular, increasing *K* increases the feature dimension of the data processed by CWSN, and increasing *h* motivates CWSN to use multi-segment features to compute long-range dependencies, but it also reduces the interpretability of the model and increases computational complexity and memory consumption.

**Figure 4:**
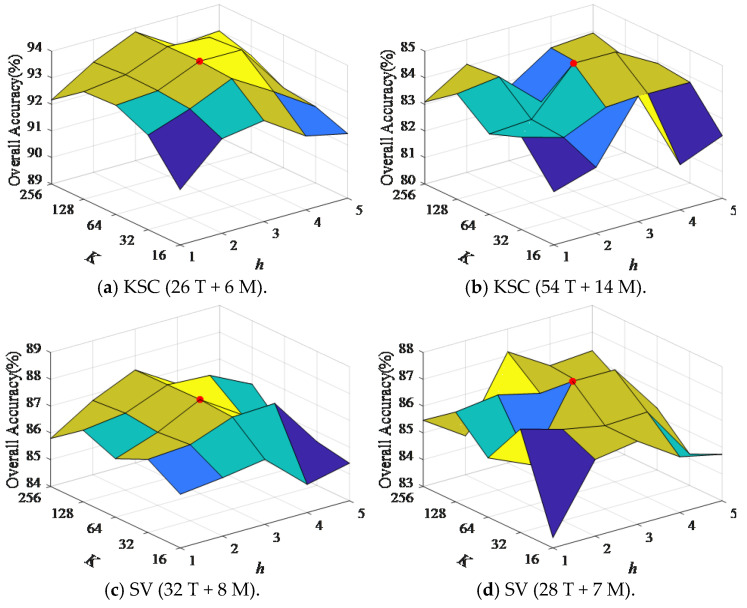
Effect of parameters *K* and *h* on the classification performance of CWSN with different numbers of mislabeled samples.

Figure 4 shows how changing the parameters *K* and *h* affects the CWSN model’s classification accuracy. Figure 4a shows that, on the KSC dataset, the OA generally increases and then decreases as the values of *K* and *h* increase, reaching the optimum when *K* = 64 and *h* = 3, indicating that the CWSN model performance is gradually optimized with changes in the values of *K* and *h*. The results in Figure 4b–d also prove that changes in values of *K* and *h* affect the performance of the CWSN model, with the optimal values of *K* and *h* being close to *K* = 64 and *h* = 3. Therefore, in this paper, based on the experimental results, *K* = 64 and *h* = 3 were set as the default parameters of the CWSN model.

### 4.3. Ablation Analysis

In this section, ablation experiments on the KSC dataset are used to evaluate the performance contribution of each module in the CWSN model. For ease of description, the convolutional part of the CWSN model is denoted as SBL (CWSN Baseline), the spectral feature extraction channel is denoted as SpeA, the spatial feature extraction channel is denoted as SpaA, and the ViT structure is denoted as TF. Table 2 shows the experimental results obtained on the KSC dataset using SBL, SpeA, SpaA, TF, and CWSN, with the highest accuracy highlighted in black bold. In terms of overall classification accuracy, the OA, AA, and Kappa metrics are the lowest for SBL and the highest for CWSN. And TF comes in second to CWSN. The three metric values of TF differ from CWSN by 2.33%, 2.34%, and 2.59% when the number of mislabeled samples is 6. The three metric values of TF differ from CWSN by 0.54%, 0.95%, and 061% when the number of mislabeled samples is increased to 13. The classification accuracy of CWSN is the most consistent across all object classes. As the number of mislabeled samples increases, the classification accuracy of CWSN fluctuates less and performs better in coping with noise.

**Figure 5:**
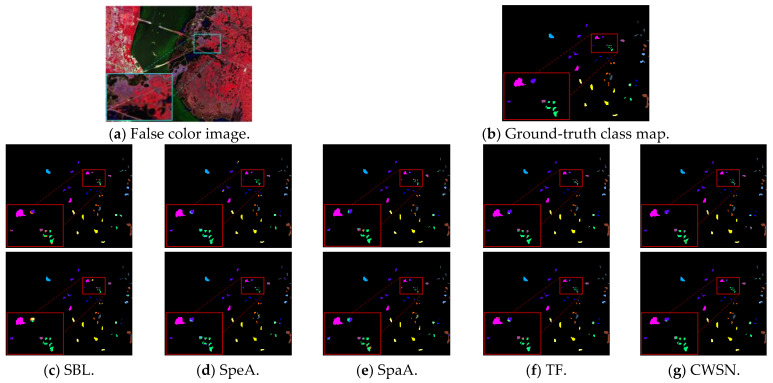
Classification maps of each component of the CWSN on the KSC dataset (first and second rows with noisy sample sizes of 6 and 13, respectively).

To analyze the classification effect of the different components in a more intuitive way, the regions in the image that are prone to misclassification were boxed and enlarged. Figure 5 shows that the classification effect gradually becomes better from left to right: the classification maps of SBL, SpeA, and SpaA have not only lower accuracy, but also have a large number of over-smoothing and misclassification phenomena. In contrast, the classification maps of the CWSN model, which combines all the core components, have neater and clearer boundaries within the region, indicating that the CWSN model is able to effectively extract the spectral and spatial features through the synergistic effect of the components, thus obtaining the best visualized classification effect.

**Figure 6:**
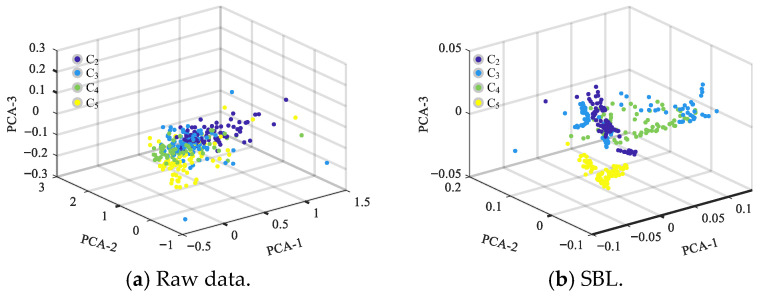

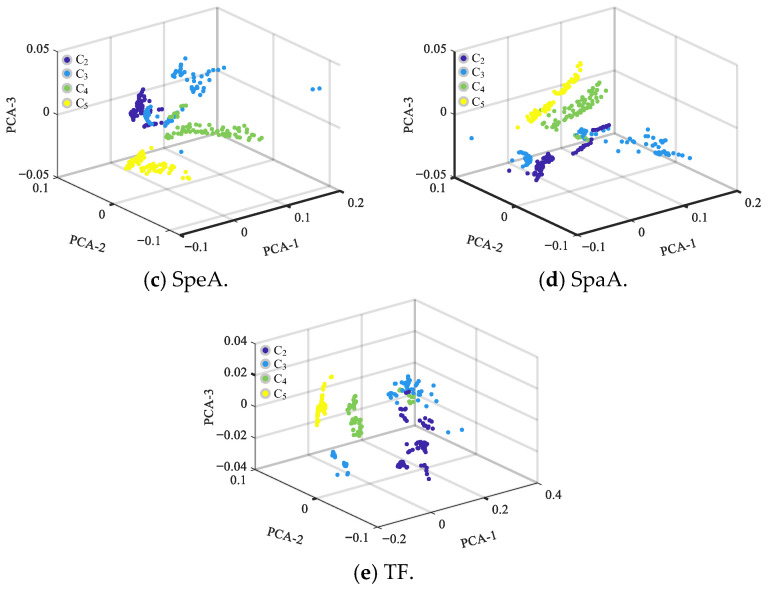
Visualization of feature extraction results for each component of CWSN on the KSC dataset.

To better demonstrate the necessity and effectiveness of each component, in addition to the individual effects above, we conducted experiments on partial combinations of components. Four classes were randomly selected from the thirteen classes of the KSC dataset, and 100 samples from each class were selected for the feature visualization of each component. The four selected feature classes were C2, C3, C4, and C5. Figure 6b shows that the sample features have low interclass separability and high intraclass similarity when the feature extraction is performed on the raw data from the four feature classes using only SBL. Furthermore, when these core components are introduced progressively to the network structure, the representation of the sample features gradually improves in separability. When the TF module is added, the sample features show high interclass separability and intraclass similarity. This indicates that these core components play a significant role in improving the accuracy of feature classification. Further analysis shows that, when the TF module is introduced, its powerful feature representation and self-attention mechanism significantly improve the model’s capacity to differentiate and consistently recognize sample features, resulting in excellent visual effects.

### 4.4. Analysis of Anti-Noise Strategies

To support the value of NSL losses, NSL was compared with existing loss functions. Table 3 shows the results of the quantitative analyses on UP. According to the data in the table, it can be seen that CE has a high sensitivity to noisy samples, which leads to its low classification accuracy on some categories, for example, the classification accuracy on C3 is only 59.27%. In terms of OA accuracy, when the numbers of mislabeled samples are 14 and 27, the RCE values are 82.68% and 82.94%, and the NSL values are 84.81% and 83.46%, respectively. This indicates that NCE has better noise immunity, but the effect on a high percentage of noisy datasets is still limited. Overall, NSL performs the best on the OA, AA, and Kappa. By combining the RCE and NCE losses, NSL has good rejection of noisy samples, which makes the model more robust and stable when dealing with noisy data.

**Figure 7:**
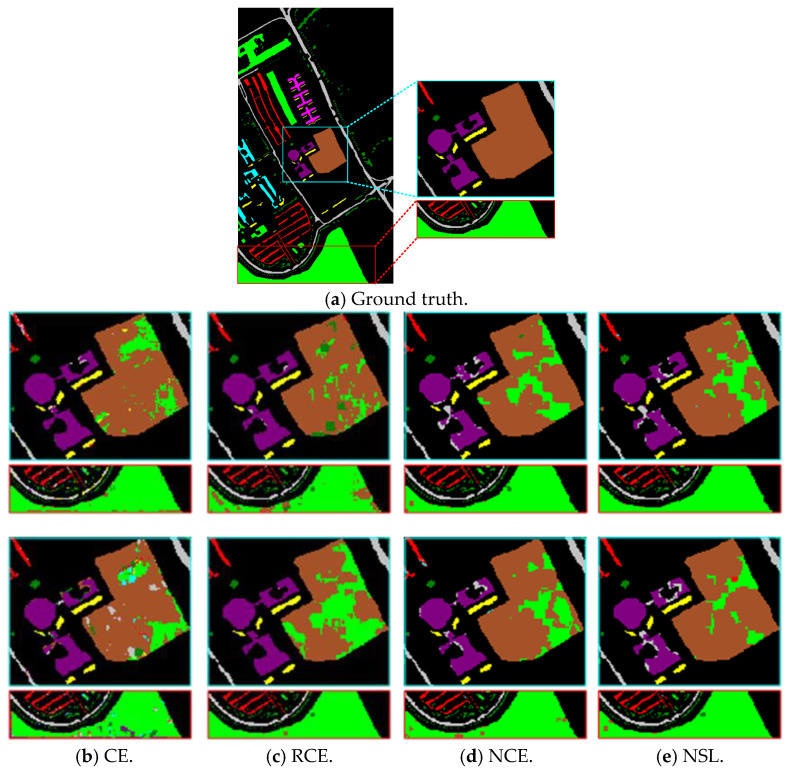
Classification results of CWSN on UP using different loss functions (54 T + 14 M in the first row and 54 T + 27 M in the second row).

Figure 7 illustrates the qualitative classification results on UP. For the two regions boxed in Figure 7a, CE performs the worst, with a lot of pretzel noise as well as unsmooth classification. RCE and NCE outperform the CE loss function in classification and show better noise immunity characteristics. Classification is the best when NSL is employed. In summary, by weighting RCE and NCR, the NSL loss function can effectively constrain the network learning and improve the ability to classify in noisy environments.

### 4.5. Computational Efficiency Analysis

On the KSC, UP, SV, and WDC datasets, this section analyses and compares the running time of CWSN with comparative methods, including mislabeled sample detection and image classification time. In this experiment, all the running time statistics experiments were conducted on the same computer. During the training process, all the datasets were trained for 100 epochs. The experimental results are shown in Table 4. As can be seen from the results in the table, compared to other methods, CWSN is not superior in terms of computational efficiency. The main reason for the high time consumption is due to the following two aspects: the Vision Transformer structure and the feature extraction process of DBLIM.

### 4.6. Comparison of Classification Performance with Low-Confidence Samples

**Figure 8:**
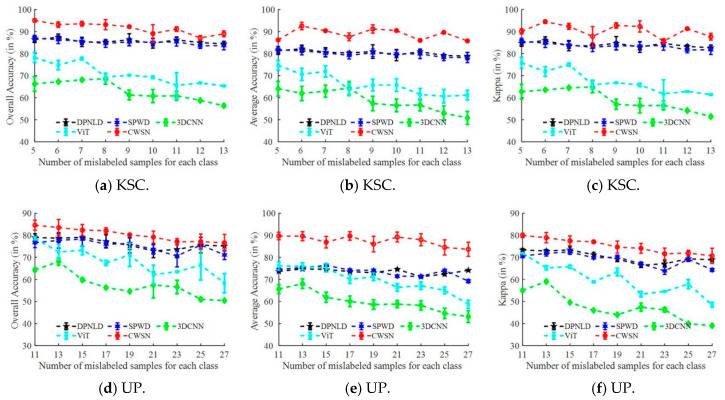

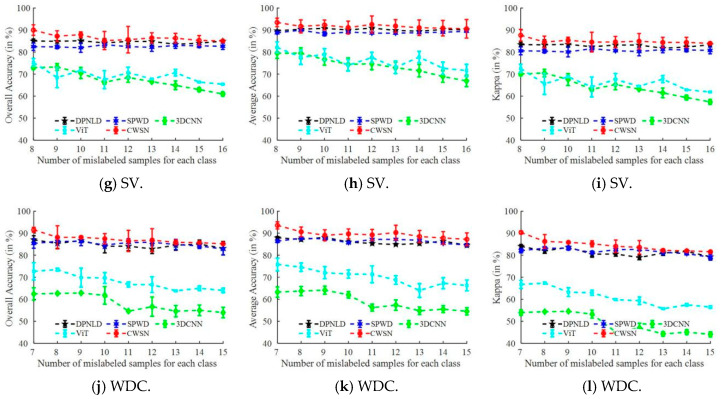
Classification accuracy of each method under different noisy sample conditions. (**a**–**c**) 26 T; (**d**–**f**) 54 T; (**g**–**i**) 32 T; (**j**–**l**) 28 T.

To investigate the robustness of the CWSN model under different numbers of mislabeled samples, DPNLD, SPWD, 3DCNN [49], ViT, and CWSN were compared on the KSC, UP, SV, and WDC datasets. Figure 8 demonstrates the classification accuracy of each method under different conditions. Taking the experimental results on the KSC dataset as an example, when there are five mislabeled samples, the OA, AA, and Kappa metrics of the CWSN model can reach 95%, 85%, and 90%, respectively, while the DPNLD and SPWD methods achieve 87%, 81%, and 86%, and 3DCNN and ViT are even lower, with an accuracy of less than 85%. When the number of mislabeled samples increased to 13, OA, AA, and Kappa metrics of the CWSN model decreased to 90%, 84%, and 89%, respectively, while the DPNLD and SPWD methods decreased to 86%, 80%, and 85%, and both 3DCNN and ViT fell below 65%. As the number of mislabeled samples increased from 5 to 13, the values of the OA, AA, and Kappa metrics generally fluctuated and decreased for each method, with the CWSN model having the smallest range of change. The above analysis is also supported by the experimental results from the other three datasets. In summary, firstly, the increase in the number of mislabeled samples has a negative impact on the performance of all classification methods, especially 3DCNN and ViT, due to the decrease in the number of clean labels, which can easily lead to underfitting of the deep learning algorithms. Secondly, the advantage of the interpretation effect of CWSN is obvious under different noise levels and datasets, suggesting that CWSN is better able to extract advanced features due to its hybrid multilevel network structure and can also be used to resist noise to a certain extent. In addition, the NSL loss function also plays an important role in noise suppression.

### 4.7. Comparison of Classification Performance with High-Confidence Samples

The anti-noise performance of deep learning algorithms comes at the expense of feature extraction and representation capability. In order to prove that CWSN is equally applicable to clean training sets, the classification performance of 3DCNN, ViT, and CWSN was evaluated on four datasets in a noise-free environment. The results are shown in Table 5. On the four datasets of KSC, UP, SV, and WDC, CWSN has the highest values of OA, AA, and Kappa metrics. For example, on the KSC dataset, the three metrics of CWSN are 3.64%, 4.07%, and 4.06% higher than those of 3DCNN, with the next best performance. On the SV dataset, 3DCNN has the best performance, but CWSN differs very little from it. Overall, the CWSN model excels in all three categories.

**Figure 9:**
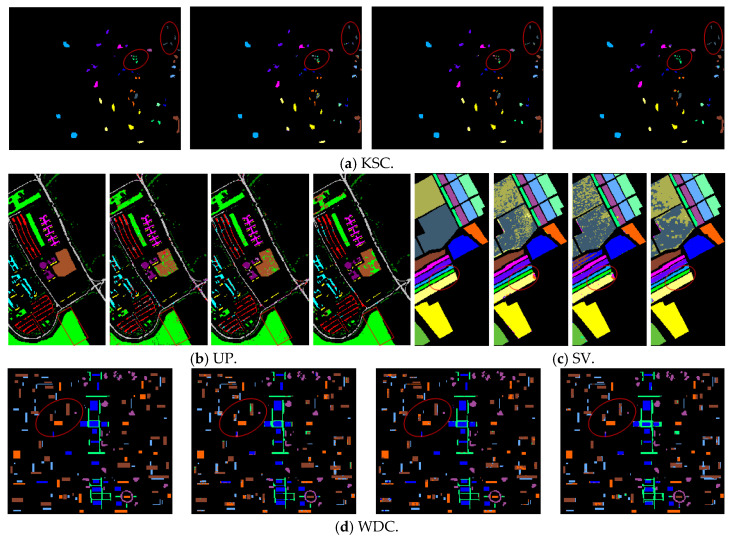
Classification maps for each method on the four datasets when using a clean sample set (from left to right in each dataset, the ground-truth, 3DCNN, ViT, and CWSN classification maps).

Figure 9 shows the classification result maps of the three methods, 3DCNN, ViT, and CWSN, on four datasets in a noise-free environment. In the KSC image in Figure 9a, the CWSN model demonstrates its strong classification ability, which is the closest to the ground truth. In contrast, the 3DCNN and ViT methods exhibit more misclassification in the boxed region of the image, resulting in a large gap between the classification results and the ground truth. The results of the dataset in Figure 9b (UP) show that the classification results of the 3DCNN and ViT methods have a large amount of salt-and-pepper noise, whereas the classification results of the CWSN are the smoothest, and are significantly better than those of the other methods, although a small amount of misclassification still exists. The classification results of the SV and WDC datasets also lead to similar conclusions. Especially in the circled part, the CWSN model performs significantly better than the other methods, and the classification results are the closest to the ground-truth images.

The reasons for this large disparity are as follows: Although ViT performs well in the image classification domain, it fails to effectively integrate spectral and spatial information when dealing with HSI. When the training data contains noisy labels or an insufficient number of labels, 3DCNN struggles to extract useful information. However, by utilizing the spectral and spatial attention mechanism as well as the Transformer structure, the CWSN model can fully capture the spectral and spatial features of HSI, improving the accuracy and efficiency of feature extraction and fusion. This multilevel feature extraction not only improves the final classification accuracy, but also enhances its robustness in complex scenes. In summary, the CWSN model also has good stability and generalization ability in the task of HSI classification under noise-free conditions.

## 5. Conclusions and Future Work

The accuracy of HSI classification under the supervised paradigm is affected by the feature extraction accuracy and sample confidence. To address the problems of deep semantic feature extraction and noisy sample detection, we propose the CWSN model, which is evaluated based on a variety of evaluation metrics in terms of model component design, classification performance, and application examples. The DBLIM adopts a lightweight dual-branch structure to enhance spatial and spectral features, with the residual connection extracting nonlinear features and mitigating the gradient vanishing in the deep network. Compared to Transformer, ViT has more advantages in image processing. So, the CViT cascade strategy combines the advantages of CNN in local feature induction and the excellent performance of Vision-Transformer in long-range correlation characterization, enhancing the robustness of the model and improving noise suppression. CWSN, which is based on a hybrid hierarchical structure, has lower computational complexity and fewer training samples required than 3DCNN, in addition to good robustness and generalizability. Extensive experimental results on five datasets demonstrate that the CWSN model outperforms other weakly supervised classification models.

When CWSN performs feature extraction and classification, the next challenge is to improve the adaptability of the ViT structure so that it can independently fully mine features of object class attributes from spectral and spatial information perspectives, thereby improving feature discrimination accuracy under small sample conditions. In addition, the Transformer structure that relies on local chunking operations has a high computational cost in computing query and key value information. By using localized objects instead of fixed-window sliding chunks, it helps to reduce computational complexity and improve the computational efficiency of the Transformer. In terms of data, spaceborne hyperspectral sensors are different from airborne sensors; currently, their spatial resolution is 30 m or more per pixel. The lower spatial resolution makes the model challenging to classify. Methods need to be explored and improved in the future, in order to achieve spaceborne hyperspectral classification application.

## Figures and Tables

**Table 1:** Settings for the number of training samples on the KSC, UP, SV, and WDC datasets.

Dataset	KSC		UP		SV		WDC	
Number of classes	13		9		16		6	
Sample setting	Setting 1	Setting 2	Setting 1	Setting 2	Setting 1	Setting 2	Setting 1	Setting 2
	26 + 6	26 + 13	54 + 14	54 + 27	32 + 8	32 + 16	28 + 7	28 + 14
Total number of samples	338 + 78	338 + 169	486 + 126	486 + 243	512 + 128	512 + 256	168 + 42	168 + 84

In sample setting, T + M represents the total number of samples for each class; T and M represent the number of correct and mislabeled samples for each class, respectively.

**Table 2:** Classification accuracy (%) of each component on the KSC dataset (Bold numbers indicate the highest accuracy).

Class	26 T + 6 M					26 T + 13 M				
	SBL	SpeA	SpaA	TF	CWSN	SBL	SpeA	SpaA	TF	CWSN
C1	**98.26**	95.34	90.82	91.73	94.54	87.89	89.89	91.69	90.58	**94.60**
C2	**97.62**	87.18	95.96	88.99	96.38	92.54	89.44	82.57	86.48	**93.03**
C3	93.57	83.61	95.76	**99.12**	92.23	**96.46**	88.62	95.23	95.54	95.54
C4	**82.22**	74.27	54.93	69.77	69.22	76.73	62.79	64.71	64.01	**86.22**
C5	91.14	83.30	88.59	**98.98**	93.00	75.28	77.03	**82.73**	80.23	76.61
C6	78.43	78.63	84.20	80.08	**95.46**	63.81	**88.55**	71.43	82.57	84.50
C7	97.10	95.71	89.32	**100.00**	95.53	96.28	91.35	**100.00**	**100.00**	84.12
C8	96.40	93.34	94.75	93.53	**97.66**	91.50	82.48	73.71	**93.79**	84.48
C9	99.18	95.40	97.04	**100.00**	97.28	**100.00**	**100.00**	92.67	95.96	**100.00**
C10	97.74	97.56	97.46	**99.91**	93.98	**99.37**	94.85	95.34	95.08	95.63
C11	**100.00**	96.44	97.14	99.83	97.23	99.65	95.69	**100.00**	93.52	96.10
C12	**96.81**	85.50	81.84	91.58	95.41	74.93	89.66	92.09	**92.43**	91.59
C13	64.14	96.14	83.40	**100.00**	**100.00**	98.53	93.97	93.28	**100.00**	**100.00**
OA	87.76	91.73	89.99	92.60	**94.93**	87.91	90.15	89.47	92.19	**92.73**
AA	89.57	89.42	90.55	91.35	**93.69**	85.69	88.02	87.34	90.01	**90.96**
Kappa	84.68	90.98	87.98	91.97	**94.56**	86.84	89.34	89.56	91.61	**92.22**

**Table 3:** Classification accuracy of each loss algorithm on UP.

Class	54 T + 14 M				54 T + 27 M			
	CE	RCE	NCE	NSL	CE	RCE	NCE	NSL
C1	88.03	84.18	**91.64**	86.49	79.09	90.09	**90.60**	89.75
C2	69.69	76.69	79.79	**86.25**	**87.68**	85.58	83.15	75.66
C3	59.27	85.85	**98.42**	88.98	70.81	90.28	87.07	**91.61**
C4	96.77	**97.05**	93.33	94.38	89.76	**95.76**	90.31	94.62
C5	**99.97**	**99.97**	99.79	99.79	95.65	99.97	**100.00**	**100.00**
C6	**84.48**	74.25	73.58	70.56	63.03	62.40	74.38	**83.54**
C7	93.60	**97.20**	96.91	96.96	86.16	94.26	**97.74**	95.42
C8	**93.39**	93.23	47.60	73.48	80.14	**90.89**	88.01	84.31
C9	97.81	**99.89**	99.51	87.21	96.33	99.38	99.92	**99.96**
OA	79.96	82.68	81.54	**84.81**	82.51	82.94	82.67	**83.46**
AA	87.00	**89.81**	86.73	87.12	83.18	89.85	90.13	**90.54**
Kappa	74.91	77.83	76.34	**80.07**	77.13	78.96	81.36	**81.53**

**Table 4:** Running time of DPNLD, SPWD, 3DCNN, ViT, and CWSN in the noise training set (s).

Method	KSC		UP		SV		WDC	
	26 T+ 6 M	26 T+ 13 M	54 T+ 14 M	54 T+ 27 M	32 T + 8 M	32 T+ 16 M	28 T+ 7 M	28 T + 14 M
DPNLD	14.19	19.30	29.09	25.73	31.05	75.36	2.26	9.49
SPWD	19.11	26.16	33.48	21.60	43.72	84.80	3.33	4.51
3DCNN	856.82	796.04	4014.01	3663.19	6429.89	6152.87	533.75	520.64
ViT	1825.77	1952.51	5439.61	4814.67	8369.64	8979.92	1105.73	1087.98
CWSN	3472.07	3040.37	8155.47	8943.99	11,816.94	11,597.85	1865.95	1982.46

**Table 5:** Classification accuracies (%) obtained by 3DCNN, ViT, and CWSN models on four datasets using the CE loss function.

**Dataset**	**KSC**			**UP**		
	**3DCNN**	**ViT**	**CWSN**	**3DCNN**	**ViT**	**CWSN**
OA	91.79	91.66	**95.43**	86.07	90.57	**91.75**
AA	89.30	89.14	**93.37**	88.62	90.91	**92.48**
Kappa	90.84	90.69	**94.90**	81.92	87.53	**90.10**
Dataset	**SV**			**WDC**		
	**3DCNN**	**ViT**	**CWSN**	**3DCNN**	**ViT**	**CWSN**
OA	**88.15**	84.46	86.72	85.47	88.96	**89.60**
AA	**93.98**	92.27	93.22	86.93	91.38	**92.01**
Kappa	**86.81**	82.73	85.24	82.08	86.41	**87.43**

## Data Availability

Data are contained within the article.
